# Towards a unified terminology for sonification and visualization

**DOI:** 10.1007/s00779-023-01720-5

**Published:** 2023-08-21

**Authors:** Kajetan Enge, Alexander Rind, Michael Iber, Robert Höldrich, Wolfgang Aigner

**Affiliations:** 1 Institute of Creative Media Technologies, FH St. Pölten, Campusplatz 1, St. Pölten, 3100 Austria; 2 Institute of Electronic Music and Acoustics, University of Music and Performing Arts Graz, Leonhardstraße 15, Graz, 8010 Austria

**Keywords:** Sonification theory, Visualization theory, Audio-visual data analysis

## Abstract

Both sonification and visualization convey information about data by effectively using our human perceptual system, but their ways to transform the data differ. Over the past 30 years, the sonification community has demanded a holistic perspective on data representation, including audio-visual analysis, several times. A design theory of audio-visual analysis would be a relevant step in this direction. An indispensable foundation for this endeavor is a terminology describing the combined design space. To build a bridge between the domains, we adopt three of the established theoretical constructs from visualization theory for the field of sonification. The three constructs are the *spatial substrate*, the *visual mark*, and the *visual channel*. In our model, we choose time to be the *temporal substrate* of sonification. *Auditory marks* are then positioned in time, such as visual marks are positioned in space. *Auditory channels* are encoded into auditory marks to convey information. The proposed definitions allow discussing visualization and sonification designs as well as multi-modal designs based on a common terminology. While the identified terminology can support audio-visual analytics research, it also provides a new perspective on sonification theory itself.

## Introduction

Designers of sonification systems can nowadays base their work on a solid foundation of research on auditory perception and several sonification techniques such as auditory icons, parameter mapping, and model-based sonification [2, 3]. Thus, a theory of sonification already has an articulated set of design constructs at its disposal [4]. However, we argue that constructs at a more basic level are missing from the current stage of scientific dialogue. This seems to be especially relevant for the design, description, and evaluation of combinations of sonification and visualization.

This article^1^ proposes *channels* encoded into *marks* that are positioned in a *substrate* as basic constructs for designing sonifications. The theoretical model is adopted from the visualization literature [5–7], where channels, marks, and spatial substrate are widely used constructs. They allow the description of the extensive design space of visualization approaches using only a small set of atomic building blocks, and have thus been successfully used as framework for guidelines (e.g., [7]), software tools (e.g., [8]), and toolkits (e.g., [9, 10]), as well as automatic recommendation of visualizations (e.g., [11–13]).

Theoretical cross-pollination between visualization and sonification is most reasonable because both fields share similar goals. While sonification is “the use of nonspeech audio to convey information” [14], visualization is defined as “the use of computer-supported, interactive, visual representations of abstract data to amplify cognition” [6].

Unsurprisingly, sonifications are often employed together with visualizations in real-world scenarios, for instance, by diagnostic ultrasonic devices. However, too little attention has been paid to the theoretical underpinnings of audio-visual data analysis approaches [15]. Such approaches essentially use both our vision and our auditory sense in combination to convey information about data. Bridging terminological barriers between the research communities is a reasonable step towards a combined design theory with compatible basic constructs and making progress in both fields.

There are, however, fundamental differences between our visual and auditory perception [15]. For example, with regard to spatial resolution, auditory perception is less accurate than visual perception [16]. Sound is an inherently temporal phenomenon [17–20] unlike vision. Therefore, adaptations of the model of channels, marks, and the substrate are needed.

This article starts with related work (Section 2) and an introduction to the constructs of the substrate, marks, and channels from visualization literature (Section 3). Section 4 investigates how equivalent constructs can be defined for the sonification domain and provides a mathematical description of auditory marks. In Section 5, we discuss analogies between sonification and visualization practice emerging from our model and analyze existing designs from sonification and visualization literature with our model. Before we conclude in Section 7, we argue for the rejection of space and frequency as substrates for sonification in Section 6.

With this article, we propose a new way to describe combinations of visualization and sonification. A terminology that uses the same basic constructs will help members of both communities with discussing their work and with combining their knowledge.

Our original paper [1] has been extended by

- A discussion of the construct of auditory channels,
- A discussion of frequency as a potential substrate for sonification, and
- A demonstration of the unified terminology by describing existing work using the adopted constructs.

## Related work

There are numerous examples of designs that combine sonification and visualization and many of them can be found via the “Data Sonification Archive” via https://sonification.design. Recently, Caiola et al. [21] analyzed 80 examples of audio-visual designs leading towards their definition of an “audiovisual design map,” meant to support the integration of sonification and visualization. Hildebrandt et al. [22] combined visualization and sonification to analyze business process execution data. Rabenhorst et al. [23] augmented a vector field visualization with sonification. Chang et al. used an audio-visual approach to explore the activity of neurons in the brain [24]. In 2003, Hermann et al. presented “AVDisplay” [25], a system for monitoring processes in complex computer network systems including both sonifications and visualizations. MacVeigh and Jacobson [26] described “a way to incorporate sound into a raster-based classified image.” They augmented a map with further dimensions through sonification.

Taken together, the abovementioned works support the notion that visualization and sonification can be combined for effective data analysis. Nesbitt introduced a taxonomy for the multi-modal design space [27–31]. He proposed essentially two ways to describe the multimodal design space, including haptic displays. The first is an extension of the reference model for visualization by Card, Mackinlay, and Shneiderman [6], which we also choose as our reference in this article. In his extended design space, Nesbitt uses space as the substrate for visual, auditory, and haptic displays. His second description of the multi-modal design space is based on three types of metaphors: spatial metaphors, temporal metaphors, and direct metaphors [31]. These categories take into account the inherent temporal structure of sound. While Nesbitt introduced a new description of the multi-modal design space, in this article, we suggest using time instead of space as the substrate of sonification and adopting the vocabulary from visualization theory, as will be argued in the following.

**Fig. 1:**
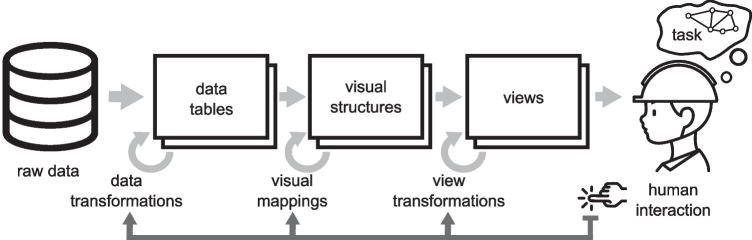
The reference model for visualization [6] introduces visual structures as an intermediate state in mapping data to visual representations (figure from [1], CC BY). Reusing the icon “engineer” by Pawinee E. from Noun Project, CC BY 3.0

Compared to visualization, sonification is a considerably younger discipline [32]. This might be one of the causes why its theoretical foundation is not as developed even though both disciplines pursue very similar goals [4]. In sonification, some of the milestones in theory development have been the “Proceedings of the 1st Conference on Auditory Display” in 1992, which were edited in the book *Auditory Display* in 1994 [33], marking the beginning of systematic research on sonification by the international community for auditory display. Barrass’ dissertation in 1997 [34] introduced task analysis, data characterization, and a case-based design method to the community. The sonification report in 1999 [14] provided an overview of the field at the time and a definition of sonification that is still widely used. Walker [35] worked on magnitude estimation and mapping-polarity of conceptual data dimensions in 2002 and Hermann [36] studied sonification in the context of exploratory data analysis. The book *Ecological Psychoacoustics*, edited by Neuhoff in 2004 [37], provides a more holistic perspective on psychoacoustics than conventional laboratory studies could offer. The design space map introduced by de Campo in 2007 [38] helps a designer decide on an appropriate sonification technique with respect to the number of data items and attributes to be sonified. Hermann’s taxonomy from 2008 [3] provides a detailed definition of sonification and auditory display in a scientific context. The *Sonification Handbook* gave another overview of the field in 2011 [2], and Worrall’s *Sonification Design* [39] put another focus on both theory and design of sonifications in 2019.

However, in 2019, Nees [4, p. 176] stated that “[...] sonification theory remains so underdeveloped that even the path to advance theory-building for sonification remains unclear.” He then refers to an article by Gregor and Jones [40] as inspiration for the development of a sonification design theory. Gregor and Jones describe eight components that any design theory should include, specifically, (1) purpose and scope, (2) constructs, (3) principle of form and function, (4) artifact mutability, (5) testable propositions, (6) justificatory knowledge, (7) principles of implementation, and (8) expository instantiation.

In this sense, our article focuses on the *constructs* of a design theory, as they are especially relevant for a combined terminology of sonification and visualization. Gregor and Jones [40, p.33] describe the constructs: “The representations of the entities of interest in the theory [...] are at the most basic level in any theory. These entities could be physical phenomena or abstract theoretical terms.” The state of the art of the eight components for a design theory of sonification is well described in the 2019 paper by Nees [4].

In our work, we intend to contribute to the development of a design theory for the combination of sonification and visualization by offering low-level constructs for the description of sonification designs. We do so by adopting some of the elaborated theoretical constructs from visualization theory for the domain of sonification. In the following section, we introduce these constructs: the spatial substrate, the mark, and the channel.

## Basic theoretical constructs in visualization theory

Since the design space of possible visualization solutions is extensive, the visualization community has worked on theoretical models to formalize design knowledge [7]. Based on Bertin’s seminal book *Semiology of Graphics* [5], many visualization models (e.g., [6, 7, 9, 11, 41]) are centered around marks as the basic building blocks of visualization techniques. In general terms, a mark is a geometric object that represents the attributes of a data object by position, color, or other visual features.

The widely adopted reference model for visualization by Card, Mackinlay, and Shneiderman [6] provides the more specific formalism needed for a transfer to the field of sonification. It dissects visualization as a pipeline of data transformations from raw data to a visual form perceived by humans. In the center of this pipeline, there are *visual structures* that consist of marks positioned in a spatial substrate and channels that encode information to the marks’ features. These visual structures are created from data tables and subsequently projected onto a view for display (Fig. 1).

### Defining visual structures

The three components of a visual structure are the spatial substrate, marks, and channels.

*Channels* such as position and color encode the information of the data table’s attributes into the visual features of the marks. Besides spatial position, Bertin [5] enumerates six non-positional channels: size, color hue, color gray scale value, shape, orientation/angle, and texture; yet further channels are possible (e.g., color saturation, curvature, motion [7]). The reference model originally refers to channels as “graphical properties” and the visualization literature contains a number of further synonyms such as “perceptual attributes” or “visual variables,” yet “channel” seems to be most widely used [7, p. 96]. Since spatial position allows very effective encoding for visual perception, the reference model conceptualizes it as a substrate “into which other parts of a Visual Structure are poured” [6, p. 26].

The *spatial substrate* is the conceptual space where marks are positioned. While it is most often a two-dimensional (2D) space, a conceptual three-dimensional (3D) spatial substrate can also be projected on a 2D view for display on a computer screen or viewed on a virtual reality device. Different types of axes and nesting mechanisms subdivide the spatial substrate.

**Fig. 2:**
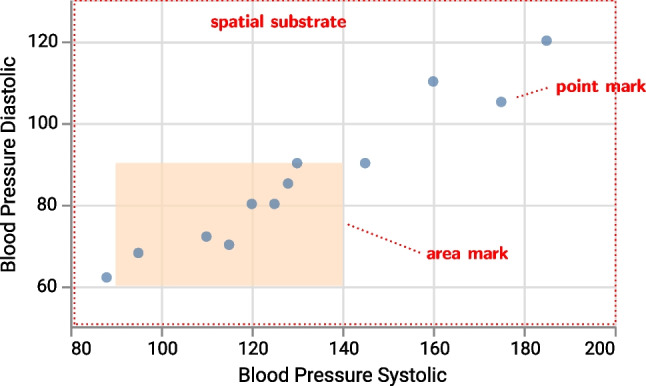
Example scatter plot with blood pressure measurements (artificial data) as *points* (0D) and a rectangle representing the *area* (2D) of normal systolic and diastolic blood pressure (figure from [1], CC BY)

The reference model distinguishes four elementary types of *marks*: points (zero-dimensional, 0D), lines (one-dimensional, 1D), areas and surfaces (2D), and volumes (3D). Marks can have as many dimensions as their containing substrate; therefore, surfaces and volumes occur only in 3D substrates. Furthermore, the visualization reference model introduces special mark types to encode connection (e.g., in a node-link diagram  ) and containment (e.g., in a Venn diagram  ). For example, the dots in a 2D scatter plot are point marks (0D) positioned along two orthogonal quantitative axes, and in the same plot, an area mark (2D) can represent a range of values along both axes (Fig. 2). The countries in a choropleth map are also area marks positioned in a geographical spatial substrate. An example of 1D marks is the line in a line plot or isolines on a geographic map.

The distinction between mark types depends not only on their visual form but also on the data object represented by the mark—whether the data object encodes information for a point in the spatial substrate, or it encodes information about some extent of the spatial substrate. In fact, the rendered marks need to have some extent in all dimensions of the spatial substrate (e.g., 2D) because an infinitely small point or an infinitely thin line would not be visible.

**Fig. 3:**
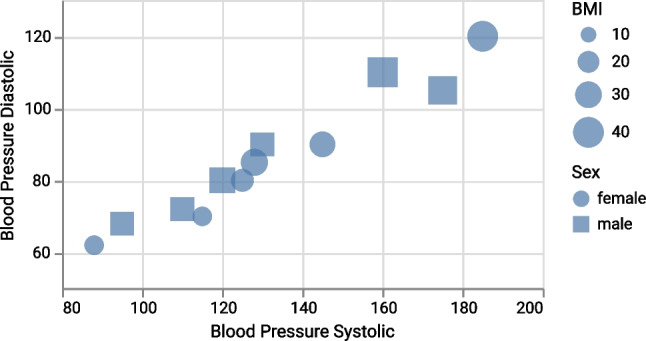
Example scatter plot (artificial data) using size and shape as two channels. Note that rectangles and circles represent *point* marks (0D) (figure from [1], CC BY)

Since the spatial extent of a point mark does not convey information per se, the mark is not constrained and can use the channel *size* to encode a data attribute. Yet another data attribute can be mapped to the channel *shape*, so that one category is shown as square and another as circle (Fig. 3). Neither the size nor the shape channel can be mapped to an area mark (cp. Fig. 2) because its spatial extent is constrained by the represented information.

Finally, these examples illustrate how the same visual form, in this case a rectangle, can represent either a data object positioned at a point with size and shape (Fig. 3) or a data object spanning an area in the spatial substrate (Fig. 2). To correctly interpret such graphics, contextual information is necessary that visualization designers need to provide via legends, annotations, or other onboarding approaches [42].

### Applying visual structures

Within this conceptual model, the design space of visualization techniques stretches over all possible combinations of marks, spatial substrates, and channels. It provides a terminology to characterize existing techniques such as the scatter plot (Fig. 2) and to invent completely new techniques. Several visualization software frameworks apply these constructs to specify the visual encoding: e.g., Tableau [8], ggplot2 [43], RAWGraphs [44], or Vega-Lite [10].

**Fig. 4:**
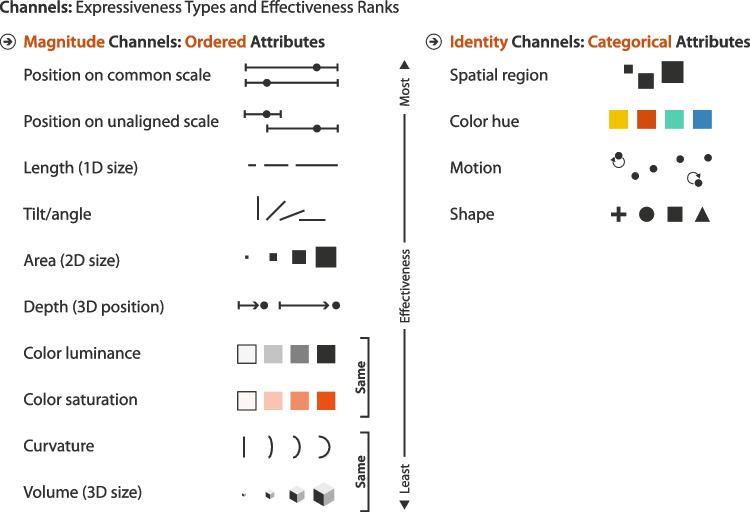
Munzner’s guideline to group visual channels into magnitude and identity channels and rank them by effectiveness [7, p. 102] (figure from “Visualization Analysis and Design” [7] by Tamara Munzner, with illustrations by Eamonn Maguire, AK Peters Visualization Series, CRC Press, 2014, CC BY 4.0.)

The use of spatial substrates, marks, and channels ensures a consistent mapping from data to visual form, and thus promotes visual pattern recognition. The resulting graphic can be read as a whole, as individual marks, and at multiple intermediate levels [5]. For example, proximity on the spatial substrate and similarity of the color channel can be perceived as Gestalt. However, not all combinations of marks, substrates, and channels result in an effective representation of its underlying data. Yet, this conceptual model helps to systematically investigate the effectiveness of the visualization’s components. For example, the experiments by Cleveland and McGill [45] found that the position channel was superior to length or angle in terms of accuracy.

Such results from empirical work can be distilled to design knowledge that is published as guidelines. For example, Mackinlay [11] ranks channels by their accuracy on perceptual tasks with quantitative, ordinal, and nominal data. Thus, he compares channels not only by their effectiveness, but also by their expressiveness. In another design guideline, Munzner [7] distinguishes *magnitude channels*, expressing quantitative or ordinal data, from *identity channels*, expressing categorical data, and ranks both by their relative effectiveness (Fig. 4). The position, size, and tilt of visual marks are conventional magnitude channels that inform about “how much of something there is”[7, p.99]. Color hue and shape are often used as identity channels, informing users about “what something is” [7, p.99]. Likewise, design knowledge is integrated into tools such as APT [11], Tableau [12], and Vega-Lite [13] for automated visualization recommendations.

Overall, marks, spatial substrates, and channels have shown to work well as a formal model for visualization techniques. We assume that these constructs lend themselves to formalizing sonification techniques as well, thus paving the way for creating audio-visual techniques for data analysis.

## Adopting the constructs for sonification

To develop a combined design theory for audio-visual analytics, it is important to use common theoretical constructs. Such constructs define the terminology necessary to discuss audio-visual techniques at a conceptual level. In this section, we adopt the theoretical constructs that have been established in the visualization community for the field of sonification. First, we generalize the three constructs “substrate,” “mark,” and “channel”: The substrate is the conceptual space on which a data representation is instantiated; it “holds” the marks. Marks are the perceptual entities of a data representation that can be distinguished by their conceptual expansion within their substrate. Channels are the parameters of a data representation encoded in a mark, carrying the information.

Next, this section investigates possible analogies for these constructs in sonification. On the one hand, in sonification, the construct of channels is relatively familiar with parameters such as loudness, pitch, or timbre [2, 35]. However, the two constructs of substrate and marks are not commonly used to describe a sonification. Since marks expand conceptually within their substrate, these two constructs are closely intertwined. As visualization uses space as a substrate, we will discuss the potentials and limitations of space and frequency as possible substrates for sonification in Section 6. However, the potential of time as the substrate for sonification has shown to be more promising.

**Fig. 5:**
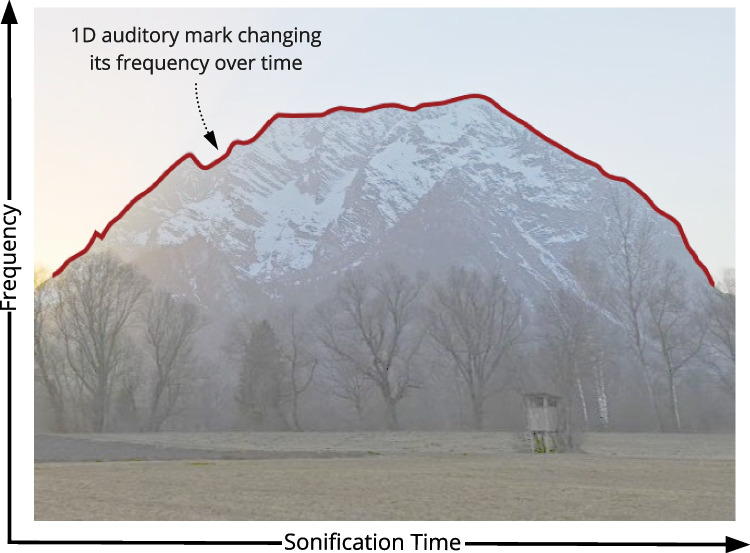
The silhouette of the mountain “Grimming” in Austria. A 1D auditory mark maps the horizontal positions of the silhouette to time, and the height of the silhouette to the frequency of a sine wave. The horizontal positions correspond to the sortable attributes *k* and the height values to the attributes *x* from Fig. 6 and Eqn. 3 (figure from [1], CC BY)

### Time as the substrate of sonification

Next to space, we have another fundamental dimension at our disposal: time. If we compare the dimensions space and time against each other, we find several arguments and analogies in support of time as the substrate for sonification.

Both time and space are physical dimensions inherently bound to our visual and auditory perception. However, with respect to sonification, spatial locatability is not necessary for the perception of a sound. When we hear a mono sound originating in front of us, we will hear it from the position of the loudspeaker. When we hear the same sound over headphones we will perceive it within our head (internalization). Our perception of the sound itself will not be altered; hence, the sonified information we perceive is consistent. Therefore, we argue that the perceived acoustic space is not inherently necessary for sonification. Time, on the other hand, is a dimension that we cannot even conceptually “switch off” while listening: A sonification that does not expand over time is not imaginable.

The opposite holds true for visualizations and space as their substrate: A visualization without spatial extent is not imaginable, while time is a dimension that can be conceptually “switched off” as long as the visualization is static (i.e., not using informative animation). Even though scanning a visualization involves eye movements at a rate between two to five saccades per second [46, p. 144–145] and analyzing a dataset is an iterative visual search process, the static visualization itself does not change over time.

Using this analogy, one can think of sounds being “positioned in time” in a sonification, just as visual marks are positioned in space. This is also supported by the fact that, with our eyes, we have a precise resolution for the relative spatial position of two visual objects, while with our ears, we have a far better temporal resolution for the relative position of two sounds. Furthermore, the temporal structure of sound is perceivable with only one ear, while generally we have to use both of our ears to detect spatial cues [16].

For these reasons, we consider time to be a suitable substrate for sonification and refer to it as the “temporal substrate.” For the temporal substrate, it is not relevant whether the sonification is passively listened to or whether somebody interacts with it. In our model, time as a dimension is always considered to be linear. The follow-up question must be how to define types of auditory marks in a temporal domain.

### Auditory marks

We know that visualization theory distinguishes its visual marks by their conceptual dimensionality, i.e., their conceptual extent within the spatial substrate. As has been shown, conceptual expansion does not have to be equal to physical expansion. Visual marks need to occupy space to become visible, even if conceptually they do not expand [5]. Correspondingly, it should be possible to distinguish auditory marks by their conceptual expansion within their substrate, time. Two more questions arise: How do we define conceptual expansion in time, and how many different types of auditory marks exist?

In visualization theory, the four mark types are “points,” “lines,” “areas,” and “volumes” [6]. They represent all the possibilities for conceptual spatial expansion from 0D (no conceptual expansion) up to 3D (maximal possible conceptual expansion). While space is three-dimensional, time is one-dimensional. Thus, we define auditory marks that are 0D (no conceptual expansion) or 1D (maximal possible conceptual expansion). We cannot define 2D or 3D auditory marks, since time does not provide a second or third dimension for the marks to unfold in. We consider an auditory mark as 0D if it *does not conceptually expand* in time, just as a visual mark that does not expand in space is 0D. If an auditory mark *conceptually expands in time*, it is considered as 1D, equivalent to the definition of a visual mark.

For better readability, whenever we speak of an auditory mark, we automatically mean a temporal auditory mark, and whenever we speak of a visual mark, we mean a spatial mark. Following this logic, audio-visual data representations can use both visual marks, positioned on the spatial substrate, and auditory marks, positioned in the temporal substrate. Next, we will formally define 1D and 0D auditory marks and provide mathematical descriptions of both types.

#### 1D auditory marks

A 1D auditory mark represents the data via its development over time. More precisely, the *temporal evolution* of a 1D auditory mark represents a dataset along one of the set’s sorted attributes. It does so by evolving its channel(s) over time according to the sort, thus representing the evolution of attributes in the dataset. We regard the 1D auditory mark as “conceptually expanded in time” as it conveys information over time. The sorted attribute has to be a key attribute. A key attribute is a unique identifier for all items in a dataset. In a table, it could be, for example, the row number. This ensures that every item in the dataset is mapped to time bijectively.

An illustrative example of such a 1D auditory mark is shown in Fig. 5 via the silhouette of a mountain as a red line. Imagine a *parameter mapping sonification* [47], conveying information about the shape of the silhouette. The sonification maps the horizontal and vertical positions of the silhouette to the temporal and spectral evolution of a sine wave: Moving along the silhouette from west to east results in rising frequency whenever the mountain has an uphill slope, and falling frequency whenever it has a downhill slope. In such a case, we speak of an auditory graph as a special version of a parameter mapping sonification [48, 49]. In this example, the sonification uses a one-dimensional auditory mark, since its channel (frequency) evolves over time according to the development of the vertical position sorted along the horizontal position in the dataset.

**Fig. 6:**
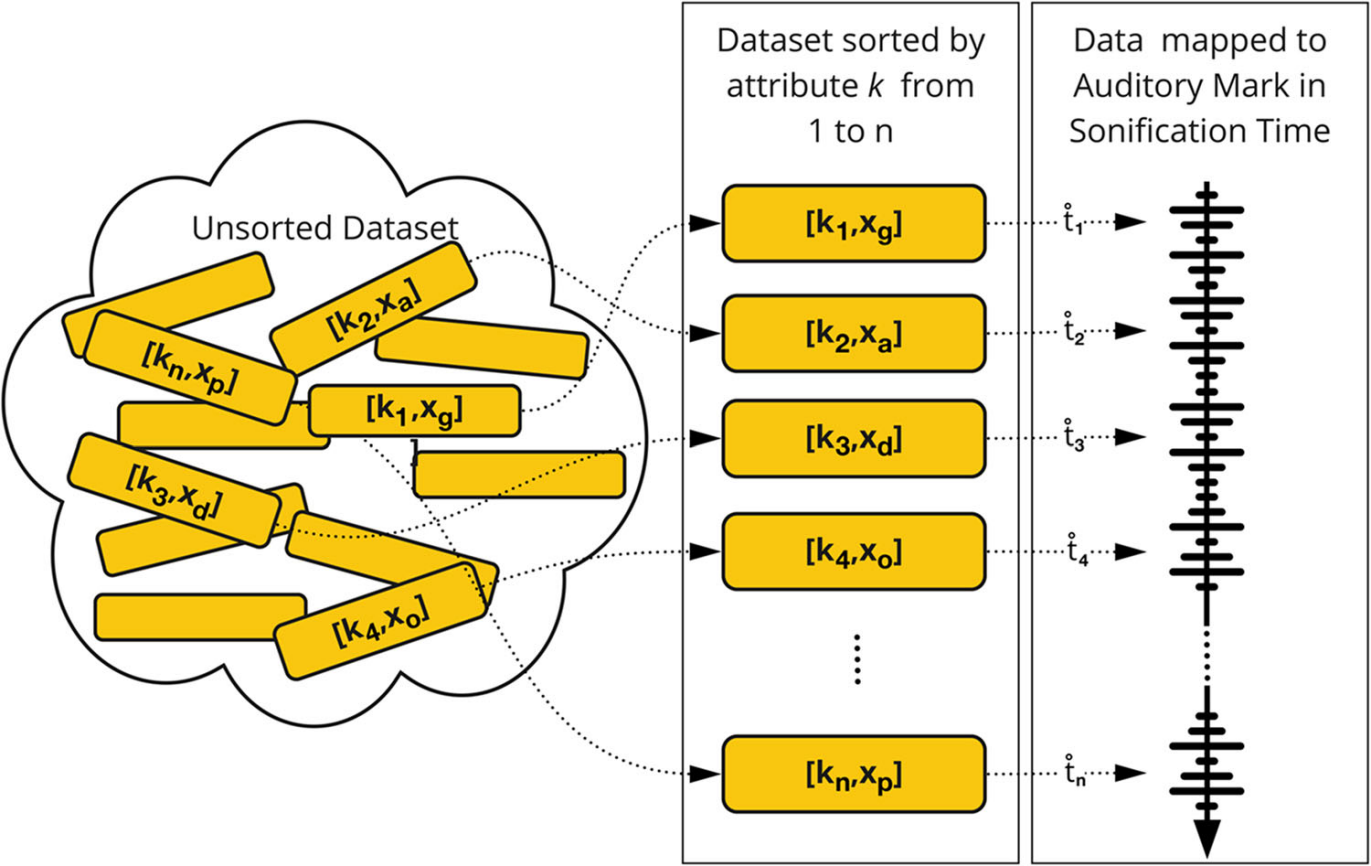
An unsorted dataset is sorted and sonified to a 1D auditory mark, evolving over sonification time (figure from [1], CC BY)

We now have defined the theoretical construct of a 1D auditory mark that conceptually expands in its substrate, in time. We still have to provide a definition of the 0D auditory mark. Every sonification has to expand in time, but not all of them convey information over time. Auditory icons and earcons, for example, are sonification techniques that convey information without an inherent dependency on developments in the data [2]. They usually inform their users about states and will be further discussed in Section 4.3

#### 0D auditory marks

A 0D auditory mark represents the data as a state in time, not as a development over time. More precisely, the *temporal evolution* of a 0D auditory mark does not represent a dataset along one of the set’s sorted key-attributes. The 0D auditory mark still needs to physically expand in time to become audible, but its temporal evolution is not bijectively representing the data over time. This can be the case if, for example, (1) there is no sortable attribute in the data, or if (2) the sorted dataset is not mapped to sonification time. For further explanation, we construct two examples.

A so-called earcon [50] can typically be described as a 0D auditory mark. The sound of a computer after an error is such an earcon and its precise temporal evolution is not informative. Instead, the meaning of such a sound has to be learned as a whole. The earcon conveys information about a state in time, not a development over time. The instant in time that the sound occurs is a channel, just like the position of a visual mark in space is a channel. The auditory mark itself conceptually does not expand in time; therefore, we identify it as zero-dimensional.

Mapping the sorted data items to frequency instead of time would also result in a 0D auditory mark. To explain this, we can reuse the silhouette example from before. The abscissa in Fig. 5 would not be the sonification time but a frequency axis, and the ordinate would not be a frequency axis but the power spectral density. In this case, the silhouette bijectively maps to the shape of a sound’s power spectral density, and the information is not encoded over time but into the spectral envelope of a static sound. This static sound is the 0D auditory mark, not evolving over time and therefore conceptually not expanded.

#### Mathematical description of auditory marks

We first want to describe the one-dimensional auditory mark in a more general mathematical way. Figure 6 shows an unsorted dataset that is first sorted and then transformed to become a 1D auditory mark. We refer to one of the attributes as *k* and to the other one as *x*. The attribute *k* is a key attribute, which means that it is a unique identifier that can be used to look up all items in a dataset [7].

1 $$\begin{aligned} k_i \ne k_j, \hspace{0.5cm} \forall i \ne j. \end{aligned}$$

To produce a one-dimensional auditory mark, *k* has to be sorted and mapped to sonification time via a strictly monotonically increasing function *f* (compare (2)). Sonification time is understood as the physical time which evolves during a sonification and is denoted as $\mathring{t}$. The ring symbol on top of $\mathring{t}$ helps to distinguish between sonification variables and domain variables. In our example, the domain variables are the horizontal and vertical positions $k_i$ and $x_i$, while $\mathring{t}$ denotes the physical time that passes while listening to the auditory mark. This convention was first introduced by Rohrhuber [51], and then developed further by Vogt and Höldrich [52]. In the silhouette example, we used the horizontal positions $k_i$ to sort the vertical positions $x_i$ from west to east.

2 $$\begin{aligned} \mathring{t_i} = f(k_i), \end{aligned}$$

We have now defined which position is mapped to which point in time. In the next step, we need to define the channel through which the mapping is realized. In our example, the channel $\mathring{c}(\mathring{t}_i)$ is the time-dependent frequency of a sine wave. Function $g(x_i)$ transforms the domain variable *x*, the vertical position, to the auditory channel *frequency* (compare [47, p. 368]). To be called sonification, this transformation must be systematic, objective, and reproducible [3].

3 $$\begin{aligned} \mathring{c}(\mathring{t}_i) = \mathring{c}(f(k_i)) = g(x_i) \end{aligned}$$

We usually deal with discrete data; therefore, some kind of interpolation between $\mathring{t}_i$ and $\mathring{t}_{i+1}$ will often be necessary. It is not necessary for $\mathring{t}_i$ to be equidistant, neither is it necessary for the interpolation to be linear. However, the mapping from the sorted attribute to sonification time has to be bijective; hence, every position on the silhouette must map to exactly one point in sonification time. Equation 4 formalizes the interpolation process with

4 $$\begin{aligned} \mathring{c}(\mathring{t}) = interp \left( \mathring{t};\{\mathring{c}(\mathring{t}_i)\}\right) , \hspace{0.3cm} \forall \hspace{0.1cm} \mathring{t}_i<\mathring{t}<\mathring{t}_{i+1}. \end{aligned}$$

Finally, the physical realization of a 1D auditory mark $\mathring{y}$ depends on the sonification time $\mathring{t}$ and the time-dependent channel $\mathring{c}(\mathring{t})$:

5 $$\begin{aligned} \text {1D auditory mark} = \mathring{y}\left( \mathring{t};\mathring{c}(\mathring{t})\right) \end{aligned}$$

A mathematical description is also possible for the 0D auditory mark. Function *g* is not mapping the attributes $x_{i}$ to sonification time $\mathring{t}$, which leads to time-independent channels $\mathring{c}$.

6 $$\begin{aligned} \mathring{c} = g(x_i) \end{aligned}$$

The comparison between (5) and (7) shows that 1D and 0D auditory marks differ in the time-dependency of their channels. The channels of 1D auditory marks are time-dependent; the channels of 0D auditory marks are not. Mathematically speaking, $\mathring{y}$ always depends on $\mathring{t}$, but $\mathring{c}$ does not have to depend on $\mathring{t}$.

7 $$\begin{aligned} \text {0D auditory mark} = \mathring{y}(\mathring{t};\mathring{c}) \end{aligned}$$

### Auditory channels

The third construct we intend to adopt from visualization theory is the *channel*. Munzner [7, p. 96] describes a visual channel as “a way to control the appearance of marks, independent of the dimensionality of the geometric primitive.” Sonification designers also control the appearance of sounds (auditory marks) using parameters such as pitch, loudness, panning/spatial position, duration, or timbre [2, 53]. The sonification community has used several terms for these parameters, such as auditory or acoustic dimensions, auditory or acoustic parameters, sound dimensions, sound parameters, sonification parameters, display parameters, or perceptual parameters [2, 14, 52–56].

With the objective of a unified design theory of combined sonification and visualization, we argue for the usage of the same terminology in both fields: visual and auditory channels. Using this terminology, it is essential to distinguish between the auditory channel in the current context and the auditory channel as a synonym for auditory perception or even the ear canal. We use the term channel with inspiration from information theory, mapping information from a source (the data) to a receiver (the human) [57].

Following the description of visual channels, we describe auditory channels as “a way to control the appearance of auditory marks, independent of their dimensionality.” We argue that also in sonification we can distinguish between magnitude channels and identity channels. Pitch and loudness are often used magnitude channels, conveying information about “how much” of something there is. Timbre (e.g., instrumentation) is a conventional identity channel, informing the listener about “what” something is [53].

While it seems reasonable to describe a parameter mapping sonification with the construct of auditory channels, it is less intuitive to use them for the description of auditory icons [58, 59] or earcons [50]. According to Gaver [59], an auditory icon uses everyday sounds to represent information that is inherently connected to that everyday sound. Deleting a document on a Mac computer, for example, triggers the sound of paper being crumpled. Earcons, on the other hand, are [60, p. 7] “abstract, synthetic tones that can be used in structured combinations to create sound messages [...].” Examples are the tone sequences played back by PCs when connecting or removing a USB drive. These series of tones do not use a single distinct acoustic parameter but still convey (categorical) information.

The essence of auditory icons and earcons is their iconic and symbolic qualities [50, 58]. Auditory icons, as icons in general, resemble their referent by an ecological connection. Symbols and earcons, on the other hand, represent their referent by a connection that has to be learned first. Everyday sounds (auditory icons) as well as tone sequences and instrumentations (earcons) could be connotated and perceived in a biased way depending on sociocultural contexts. Nevertheless, independently of such potential biases, we argue that both techniques generally use identity channels like they are described by Munzner [7].

The recording of an everyday sound such as a bird chirp can be used as an auditory icon, being interpretable due to its ecological connection to our memory of bird sounds. We identify an audio recording of a bird sound as resembling a bird, just as we identify the visual icon of a bird because it resembles the shape of a bird. Following this logic, auditory icons use an identity channel constructed from the timbre of the sound. Based on the definition given by the Acoustic Society of America, Pratt and Doak [61] refine the term timbre as "that attribute of acoustic sensation whereby a listener can judge that two sounds are dissimilar using any criteria other than pitch, loudness or duration." To think of timbre as an identity channel is also supported by a connection between “color” and “timbre” in the German language. The German word for timbre is “Klangfarbe,” which can be literally translated to “sound color.” Hence, the German language enables us to differentiate between “colors of sounds” by using a term that typically describes the acoustic qualities of instruments. It is a common practice in sonification to use different timbres (e.g., different instruments) to differentiate between items or attributes of data.

Both in visualization and in sonification, marks can combine identity channels and magnitude channels to encode more attributes. A visual point mark can use color hue as identity channel and size as magnitude channel, and in sonification an auditory mark can combine the timbre of an oboe (identity) with variable pitch (magnitude). While an auditory icon is inherently using an identity channel, it can still be parameterized with a magnitude channel, as shown, for example, by the sonification of planetary data of Elmquist et al. [62]. In such a case, an auditory icon would use for example the loudness as an additional magnitude channel to convey continuous data.

Now that we have discussed the three constructs of substrates, marks, and channels, we will explore analogies between visualization and sonification and describe examples from the literature using the terminology we have found.

## Analogies and examples

**Table 1:** Substrates, mark types, and channels

Domain	Substrate	Mark types	Possible channels
Visualization	Space	0D: Point	position, size, color hue,...
		1D: Line	
		2D: Area	
		3D: Volume	
Sonification	Time	0D: State in time	pitch, loudness, timbre,...
		1D: Development over time	

Using time as the substrate of sonification and defining marks to conceptually expand in time reveals several analogies between visualization theory and sonification. First of all, the two domains use the two most fundamental dimensions in physics, space and time, as their substrates. Table 1 shows substrates and mark types for both domains in a compact form. An analogy shows itself regarding the restrictions for a mark’s expansion. The size of a point mark does not have to be informative, so it could expand freely in size, without changing its meaning. A line mark, on the other hand, cannot change its length without changing its meaning. In our temporal definition of 0D and 1D auditory marks, we see a similar situation: A 0D auditory mark is free to expand in time, without changing its meaning, but a 1D auditory mark is not. Its duration is tied to the amount of data to be sonified. The position and size of a visual mark can be used as channels. In sonification, the instant in time and duration of an auditory mark can be channels. However, both in visualization and sonification, these parameters do not define the type of a mark. The type of mark depends on the conceptual expansion in their substrate. It is another analogy between visualization and sonification that information can be perceived on two levels: on the one hand from the appearance of individual marks, and on the other hand from Gestalts [63] that form perceptual artifacts through a group of marks with related channels. The correlation of two datasets resulting in a diagonal scatter plot is a typical example for a Gestalt in a visualization. A rhythmical pattern or a harmonic structure can be perceived as an auditory Gestalt in a sonification. Furthermore, both in visualization and in sonification, a gradual transition takes place from the sum of many 0D marks to a single 1D mark. In visualization, the best example is a dotted line: Even if every dot could have individual meaning, the Gestalt of the dots suggests a line phenomenon. The same applies to sonification and auditory perception. A violinist, to give an example from the field of music, can play a melody with the note transitions tightly tied together ("legatissimo"), or play each of them short and strictly separated ("staccato"). In both cases, a listener will recognize the tone sequence as one unit, as one Gestalt. In visualization, the different marks are perceived as individual entities, as objects with visual features. This is also reflected by the way we generally perceive our visual surroundings as humans. Bregman used the example of a green dog: We would not separately perceive a dog and the attribute “greenness”, i.e., the attribute belongs to the object [64]. He also states that “the stream plays the same role in auditory mental experience as the object does in visual” [64, p. 11]. Basically, an auditory stream is perceived to be originating from one sound source. To design effective sonifications, it is therefore necessary to be well informed about the effects that influence our perception of auditory streams.

Both in visualization and in sonification, we can define channels that encode information into the marks and can distinguish between identity channels and magnitude channels. Last but not least, just as visualization needs to deal with spatial clutter, sonification needs to deal with temporal masking.

We now want to discuss existing visualizations, sonifications, and combinations using the model of substrates, marks, and channels. These specific cases have been chosen because they give an overview of the design space that can be described and analyzed with our unified terminology.

*Examples from the visualization domain*

1. Example 1: **Node-link network diagrams** with force-directed placement [65] combine 0D point marks for network nodes with 1D line marks for their connections. An algorithm places the point marks by simulating physical forces that move connected nodes towards and unconnected nodes away from each other. In contrast to a scatter plot (Fig. 2), the position of point marks in the spatial substrate does not directly encode data attributes. Yet, the resulting placement is often effective in indicating network clusters by their proximity of marks in the spatial substrate, although cluttered areas can also be due to artifacts [7, p. 204]. Additional data attributes can be encoded with the color, size, and shape channel of point marks, as well as the color, width, and dashing of line marks.
2. Example 2: **Parallel coordinates** [66, 72] represent multivariate data as 1D line marks. On the spatial substrate, one vertical axis for each attribute is placed in parallel across the available horizontal space. The line marks, actually polygonal paths, connect the positions encoded by attribute values between adjacent axes. In addition, color hue can be used as an identity channel. The resulting plot can provide overview of multiple attributes and indicate correlation between adjacently placed attributes.
3. Example 3: The **treemap** [68] represents hierarchical data using nested rectangular area marks (2D). An algorithm iteratively divides the available spatial substrate into rectangles while mapping the size of each rectangle to an attribute summed up from the contained items. Treemaps can be applied for stock market data with stocks hierarchically grouped by sector. The marks use the size channel for market capitalization and the color channel for the relative change in stock price [69, 70].

*Examples from the sonification domain*

1. Example 1: A conventional **auditory graph** [48, 71] translates the visual representation of a linechart to an auditory representation by using a *one-dimensional auditory* mark in the temporal substrate. The auditory channel *pitch* conveys information about the data while the auditory mark evolves over time. This example shows a direct translation of a one-dimensional visual mark into a one-dimensional auditory mark by translating horizontal and vertical spatial position into temporal position and pitch.
2. Example 2: Baier et al. [72] used 0D auditory marks on the temporal substrate to encode **information about EEG signals**. To do so, they used several different auditory channels such as timbre, pitch, and duration, mapping signal parameters such as the duration between peaks in the EEG signals to auditory channels. The sonification can be listened to via their supplementary material [73].
3. Example 3: Bywater and Middelton **sonified amino acid sequences** “as a string of musical notes with sound qualities that reflect the properties of these residues” [74, p. 18]. They used 0D auditory marks (“musical notes”) in the form of marimba sounds and placed them equally distributed on the temporal substrate. Pitch was used as an auditory channel (“sound qualities”) to convey information about amino acid values in the studied sequences. The authors state that they would use other channels like timbre, dynamics, and articulation in future investigations.

*Examples from combined designs*

1. Example 1: Enge et al. [75] presented **SoniScope**, a tool that combines a visual scatterplot with interactive parameter mapping sonification. The visualization uses 0D point marks in the spatial substrate, using the channel *position* to communicate two of the data attributes. The sonification displays a third and non-visible attribute with 0D auditory marks (short marimba sounds) positioned in the temporal substrate, using the auditory channel of pitch.
2. Example 2: **Listen To Wikipedia** [76] is a website built by Stephen LaPorte and Mahmoud Hashemi enabling users to monitor changes to Wikipedia in real-time through both visualization and sonification. Whenever someone edits Wikipedia, the tool displays a 0D visual mark somewhere on the spatial substrate using the visual channels of size and color. The size encodes the size of the edit, and the color encodes whether the edit was done by an automated bot (purple), an unregistered (green), or a registered user (white). The channel *timbre* of the sounds (identifying either a bell or a string instrument) is used to communicate added (bell sounds) or removed (plucked string sounds) content on Wikipedia. The channel *pitch* again encodes the size of the edit, representing larger edits with lower pitch.
3. Example 3: Rönnberg and Johansson [77] combined a **parallel coordinates visualization with a parameter mapping sonification** to investigate the potential of sonification for the exploration of dense and visually cluttered areas. The visualization used one-dimensional line marks on the spatial substrate, encoding information via the visual channels color and position. The sonification used one-dimensional auditory marks in their temporal substrate, representing the densities of two data clusters via the auditory channel of volume of two synth sounds. The two synths represented two data clusters via the identity channel of pitch.

## Reflections on space and frequency as potential substrates for sonification

While our model uses time as the substrate for sonification, we want to discuss two other parameters especially relevant to sonification: space and frequency. Both of them come to mind when we search for a concept that can be described as “the container” of sonification. We now want to reflect on our decision to not model space and/or frequency as substrates for sonification.

### Why space is not the substrate of sonification

The ability to spread over both time and space is an essential attribute of sound. In regard to the concept of spatial substrates in visualization it may seem self-evident to assign space equally as a substrate in the sonification domain. Spatial substrates in visualization are characterized by their dimensionality. In most cases, the spatial substrate is two-dimensional, like a piece of paper or a computer screen. Three-dimensional substrates can be used in virtual reality applications or conceptually via a projection to a conventional screen. Such two- or three-dimensional spatial substrates can contain zero- to three-dimensional visual marks. In the field of audio reproduction, we commonly speak of mono, stereo, surround, and 3D reproduction of signals, thus providing the dimensionality that is required as a precondition to qualify as an equivalent to the concept of a spatial substrate in visualization.

Following this rationale, a spatially 0D auditory mark corresponds to a point mark in visualization and could be rendered using a single loudspeaker at a specific location. A spatially one-dimensional auditory mark would correspond to a line mark in visualization. Such a mark would convey different auditory information from the different spatial positions on the stereo panorama. Technically, this could be displayed with a stereo speaker setup or with a line of speakers positioned next to each other. 2D and 3D auditory marks would then be defined accordingly and could be rendered with respective surround or 3D audio systems (such as Ambisonics [78]).

What at first sight seems to be a perfectly matching analogy reveals major drawbacks at closer analysis. Spatial substrates in visualization provide clearly determined and delimited environments. Marks can be uniquely perceived and identified within these substrates. The perception of sound, however, relies heavily on psychoacoustic phenomena as they have been described by Blauert [16], Fastl and Zwicker [79], and Bregman [64]. For instance, for the stereo projection of a sound source, we utilize so-called phantom sources composite of sonic contributions of a left-hand (−30°) and a right-hand (30°) loudspeaker in relation to a listener in order for them to be perceived at specific positions between the two speakers. Even a slight turn of the listener’s head could alter the localization of the sound and change its perceived timbre. Besides the impact the coherence of sonic signals has on their localizability, overlaying sounds are also often indistinguishable for listeners, perceptually amalgamating to one compound sound. Psychoacoustic effects such as the precedence effect also contribute to the unreliability of auditory spatial perception.

Furthermore, according to Kubovy and Van Valkenburg, space is not central for the formation of auditory objects as it is not relevant from *where* a sound approaches us, but *what* sounds. In their ‘Theory of Indispensable Attributes,’ they state that it is not the direction that helps us identify an auditory object, but its temporal and spectral properties [20, 80].

Considering these ambiguities, we argue that auditory space does not qualify as a spatial substrate in analogy to its visual counterpart.

### Why frequency is not the substrate for sonification

Kubovy’s and Van Valkenburg’s work on indispensable attributes [20, 80] inspires one to think about pitch or frequency as potential substrates for sonification. Kubovy et al. plausibly argue for time and frequency as two indispensable attributes of auditory objects [20, 80]. In their original paper [20], the authors mistakenly talk about “pitch” but corrected the wording later to “frequency” [81]. They essentially state that “a *perceptual object* is that which is susceptible to figure-ground segregation” [20, p. 102] and that “an attribute (or dimension) is defined as indispensable if and only if it is a prerequisite of perceptual numerosity” [20, p. 108]. In a much earlier publication [82], Kubovy argued for pitch as a medium and a potential equivalent of space in audition. He refers to Attneave and Olson [83] with the example of a pitch-shifted melody keeping its perceptual identity.

We argue, on the other hand, that to be considered as a substrate of visualization or sonification it is relevant that a dimension enables translation-invariant placing of marks. Hence, a mark that is placed at different positions of its substrate should appear identical. It is not enough for an auditory mark to "keep its perceptual identity" like a pitch-shifted melody would, it should appear identical.

It is a quality of space that a visual mark does not change its individual appearance if it has another position on the spatial substrate. A red point in a scatter plot looks the same whether it is in the lower left corner or the upper right corner of the substrate. It conveys different information but its individual appearance is not altered by a shift in position. In search of an analog concept in sonification, we are looking for a substrate that offers the same quality to auditory marks. While time offers this quality (a sound that is only played back later will have the same individual appearance), frequency or pitch do not. A change in frequency or pitch changes the individual appearance of any sound. We want to discuss this phenomenon with two brief examples: a musical melody and everyday sounds. There are two possibilities for shifting a sound in the spectral dimension: pitch shifting or frequency shifting. A melody indeed can be transposed and still be “the same” melody, but only if the transposition happens with respect to the pitch of the individual notes. If one would change the frequencies of all the notes in a melody by a constant value, the melody would change and could not be recognized.

We humans have learned to recognize environmental sounds by listening to them over and over. That is essentially what the sonification technique of auditory icons uses to convey information to us. If one of those auditory icons would be shifted to a totally different frequency range, we would lose our environmental connection to that sound and most probably would not recognize it anymore. In such cases, even the perceptual identity of a sound would be lost.

Space and time are two dimensions that have no physical borders to our perception, while frequencies below 20 Hz and above 20kHz cannot be perceived by humans. It should be able to place an auditory mark anywhere in its substrate without losing the ability to perceive it as humans.

Due to these arguments, in our model, we do not think of frequency or pitch as adequate pendants for the spatial substrate.

## Conclusion and future work

This paper provided an overview of three fundamental theoretical constructs from visualization theory and adopted them for the field of sonification. One is the spatial *substrate*; hence, the space a visualization uses to place visual entities on. These visual entities are called *marks*; they are positioned in the spatial substrate and have visual *channels* such as size or color encoded into them. Our work shows that time qualifies as the substrate of sonification; we, therefore, call it temporal substrate. Just as visual marks have positions in space, auditory marks have positions in time. Auditory marks use auditory channels to encode information about their identity or their magnitude. We also investigated the possibility to use space or frequency as potential substrates for sonification but rejected the models due to several drawbacks. With time as the substrate of sonification, we discussed emerging analogies between sonification and visualization theory and showed how our model can be used to describe existing designs.

The possibility to use consistent theoretical constructs for the description of audio-visual data analysis techniques fosters mutual understanding and can help the visualization and sonification communities with the further development of a combined design theory. The identified constructs proved to be useful for the authors of this article in the development of two audio-visual analytics approaches: one for scatterplots [75] and one for parallel coordinates [84]. We found the common language helpful to efficiently discuss ideas while minimizing misunderstandings between the visualization and sonification experts in our team. Furthermore, our work introduces new terminology to systematically describe sonification designs and could also feed back into visualization theory concerning the temporal description of data visualizations. One strategy to evaluate the practical usability of the identified theoretical constructs would be to conduct a systematic review of cases from the literature, similar to the recent work by Caiola et al. [21].

In our future research, we will continue with the design, implementation, and evaluation of combined designs of sonification and visualization, using the theoretical underpinnings of the presented unified terminology. We will investigate how different visual and auditory channels can be combined in corresponding or complementary ways to help users explore their data. One specific next step is to tackle the known challenges of parallel coordinates, i.e., visual clutter, outlier detection, and comparability of non-adjacent axis [85, 86] with sonification. Furthermore, we will use our concept and framework of SoniScope [75] to test different combinations of visualization and sonification. Thus, we will proceed to testable propositions as another component of a design theory according to Gregor and Jones [40].

While a fundamental discussion of the possibilities for combined audio-visual designs and suggestions for novel mappings is out of the scope of this article, we want to emphasize the need for future research regarding these questions. To design expressive audio-visual displays, it will be necessary for our community to study and consider cross-modal effects on the human perception of data representations as well as Gestalt- and auditory streaming phenomena. We expect our unified terminology to support the description and communication of future guidelines in such a way that both communities can contribute to the development of an audio-visual design theory.

## Footnotes

1 This article is an extended version of our contribution to the proceedings of the 2021 Audio Mostly Conference *It’s about Time: Adopting Theoretical Constructs from Visualization for Sonification* (https://doi.org/10.1145/3478384.3478415) [1]
